# Antithrombin III concentrate therapy may have an efficacy in sepsis

**DOI:** 10.1186/cc11998

**Published:** 2013-03-19

**Authors:** N Saito, M Takeda, T Harada, R Moroi, M Namiki, A Yaguchi

**Affiliations:** 1 Tokyo Women's Medical University, Tokyo, Japan

## Introduction

Antithrombin III (AT III) has been known to contribute to anti-inflammatory response as well as its anticoagulation. Our previous study showed AT III deficiency happened in the early stage of sepsis with no relation to DIC status. However, whether AT III concentrate is a beneficial therapy or not for septic patients is controversial. Our hypothesis is that AT III concentrate may have efficacy as anti-inflammation for sepsis.

## Methods

From January 2009 to December 2011, adult septic patients, whom were given AT III concentrate in our medico-surgical ICU, were included in this study. AT III concentrate was administered 30 to 60 U/kg intravenously every 24 hours for 3 days in the patients with DIC status and AT III deficiency. Between before and after the AT III concentrate therapy, WBC (/mm^3^), CRP (mg/dl), platelet (×10^4^/μl), PT (seconds), fibrinogen (mg/dl), FDP (μg/ml), SOFA score and DIC score were evaluated. Values are expressed as mean ± SD. Data were analyzed by Wilcoxon signed-rank test. *P <*0.05 was considered significant.

## Results

There were 85 patients (52 men, 33 women; age range 19 to 92 years (mean 69.0 ± 16.9)), and the mortality rate was 27.1% and APACHE II score was 20.1 ± 7.6. WBC, CRP, PT and SOFA score were significantly improved after AT III concentrate therapy (13,176 ± 9,400 vs. 11,693 ± 7,089, *P *= 0.011, 15.9 vs. 13.3, *P *= 0.0015, 16.5 ± 4.4 vs. 15.9 ± 5.9, *P *= 0.0045, and 9.7 ± 3.8 vs. 8.6 ± 4.6, *P *= 0.01, respectively). Platelet was significantly decreased (11.7 ± 9.7 vs. 10.6 ± 9.0, *P *= 0.028), while there were no significant differences in fibrinogen, FDP and DIC score (387.3 ± 202.6 vs. 381.8 ± 163.2, *P *= 0.088, 36.9 ± 52.7 vs. 28.8 ± 39.9, *P *= 0.059, and 2.9 ± 1.6 vs. 2.6 ± 1.8, *P *= 0.25, respectively) after the therapy. One week after the therapy, platelet and DIC score were significantly improved compared with before the therapy (15.6 ± 10.0, *P *= 0.0036 and 1.8 ± 1.9, *P *= 0.0041).

## Conclusion

In the patients with septic DIC, WBC, CRP and SOFA score were immediately improved after the AT III concentrate therapy, while platelet and DIC score were improved later. AT III concentrate may also contribute to anti-inflammatory for septic DIC with anticoagulation.

